# Thrombocytopenia with absent radii syndrome with delayed presentation of thrombocytopenic episodes: a case report

**DOI:** 10.1097/MS9.0000000000000506

**Published:** 2023-04-11

**Authors:** Riffa Alassri, Nafiza Martini, Razan Othman, Hazem Kamil, Jaber Mahmoud

**Affiliations:** a Faculty of Medicine, Hama University, Hama, Syrian Arab Republic; b Stemosis for Scientific Research; c Faculty of Medicine, Damascus University, Damascus, Syrian Arab Republic

**Keywords:** case report, congenital disorder, cow’s milk protein allergy, thrombocytopenia, TAR syndrome

## Abstract

Bilateral radii aplasia with the presence of both thumbs and episodes of low platelets count are the main characteristics of thrombocytopenia with absent syndrome, which is an unusual birth disorder, with an incidence of 0.42\100 000 population.

**Case presentation:**

The authors reported a case of a 6-month-old baby girl who experienced thrombocytopenia at the age of 6 months for the first time, as a result of cow’s milk being introduced for 45 days with chronic diarrhea and growth failure. She had a deviation of the axis of the hand laterally, and a bilateral absent of radii with the presence of both thumbs. In addition, she had abnormal psychomotor development, marasmus case manifestations.

**Conclusions:**

Our aim in publishing the current case report is that clinicians caring for patients with thrombocytopenia with absent syndrome will be aware of the myriad of complications, which may occur in the other organ systems so that they can promptly diagnose and treat any associated abnormalities.

## Introduction

Highlights

- Thrombocytopenia absent radius (TAR) syndrome is a rare autosomal recessive congenital disorder, characterized by bilateral absence of the radii but both thumbs are presence, and thrombocytopenia that is generally transient.
- We reported a case of a 6-month-old baby girl with TAR syndrome who experienced thrombocytopenia at the age of 6 months for the first time, as a result of cow’s milk being introduced for 45 days. In addition, she had abnormal psychomotor development, Marasmus case manifestations.
- Clinicians caring for patients with TAR syndrome will be aware of the myriad of complications which may occur in the other organ systems so that they can promptly diagnose and treat any associated abnormalities.

Thrombocytopenia absent radius (TAR) syndrome is a rare autosomal recessive congenital disorder, characterized by bilateral absence of the radii but both thumbs are presence, and thrombocytopenia that is generally transient^1^. Thrombocytopenia, usually less than 50 platelets/nl (normal range: 150–400 platelets/nl)^2^, it is congenital or develops within the first few weeks to months of a child’s life. Individuals with TAR have low numbers of megakaryocytes and frequently present bleeding episodes in the first year of life, which diminish in frequency and severity with age^3^.

However, almost two-thirds of individuals with TAR syndrome have a cow’s milk allergy, and the introduction of cow’s milk can cause an episode of thrombocytopenia.

Other anomalies of the skeleton, cardiac anomalies, and genitourinary system can occur. The skeleton anomalies vary from hypoplasia of the radius to phocomelia of the upper limbs, with or without lower limb defects^1^.

TAR syndrome is a complex genetic disorder caused by the compound inheritance of a rare null allele and a low-frequency hypomorphic noncoding variant in the RBM8A gene^4^.

Our aim in publishing the current case report is that clinicians treating patients with TAR syndrome will be aware of the myriad of complications, which may occur in the other organ systems so that they can promptly diagnose and care of any associated abnormalities.

The work has been reported in line with the Surgical CAse REport (SCARE) 2020 Criteria^5^.

## Case presentation

A 6-month-old Syrian baby girl was referred to Children’s Hospital in Damascus with a 45 days history of chronic diarrhea and growth failure.

The patient was born at term by C-section delivery in a result of dystocia to a 14-year-old woman. The pregnancy was uneventful with no history of maternal illness, except for mention of exposure to a simple radiography without specifying trimester. The history of perinatal is unknown by parents.

The patient was fed breast milk during the first 3 months of life, without any weight gain. Many types of milk were added to the diet, including cow’s milk. Diarrhea improved with cow’s milk and then returned.

She had an abnormal psychomotor development, she is unable to hold her head, or sit with help, there is no dorsoventral or ventrodorsal inversion, although she laughs, babbles, and recognizes her mother.

On physical examination: The weight at birth was 3000 g and present weight is 3000 g, length: 68 cm, mid-upper arm circumference: 9 cm, and head circumference: 39 cm.

**Figure 1:**
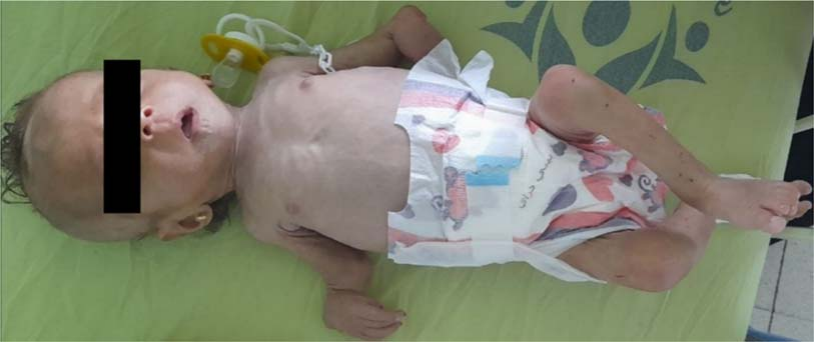
Physical examination shows loose skin folds, baggy pants sign, visible loss of fat, and muscle which suggest a Marasmus case.

Loose skin folds, the baggy pants sign, visible loss of fat, and muscle were noticed, suggesting a Marasmus case (Fig. 1). In addition to deviation of the axis of the hand laterally, bilateral absent of radii with both thumbs was noticed.

No deformities were shown in the lower limbs. The anterior fontanel is slightly open, with the existence of a depressed nasal bridge, and a prominent forehead.

Her abdominal and cranial ultrasonography were normal.

Initial laboratory investigations gave the following results: hemoglobin: 11 g/dl, mean corpuscular volumeMCV: 82fl, white blood cell: 32 200/mm^3^, neutrophils: 54%, lymphocyte: 37%, and thrombocytobenia with platelet count of 42 000/mm^3^.

There was an increasing Immunoglobulin IgA and IgM, IgA:1.01g/l, IgM:1.23g/l.

Urinalysis revealed that the urine contained reduction bodies, and stool examination showed increasing fatty substances.

The peripheral blood smear showed hypochromic, small cell size, leukocytosis, and lymphatic preponderance with some nontypical lymphocytes and thrombocytopenia.

A bone marrow biopsy was performed and revealed a normal cellularity, myeloid series: 60% shift to the left with eosinophilia, erythrocyte: 30%, hypo-megakaryocyt megaloblastic findings without megacaryoblast.

Parents refused to perform genetic testing.

**Figure 2:**
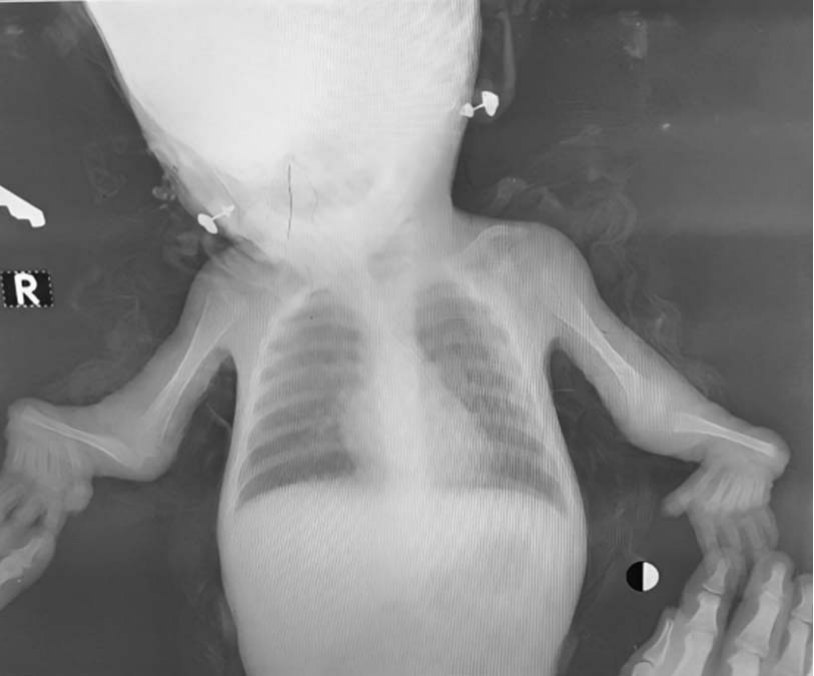
X-rays of the upper limbs demonstrated bilateral absence of the radii.

**Figure 3:**
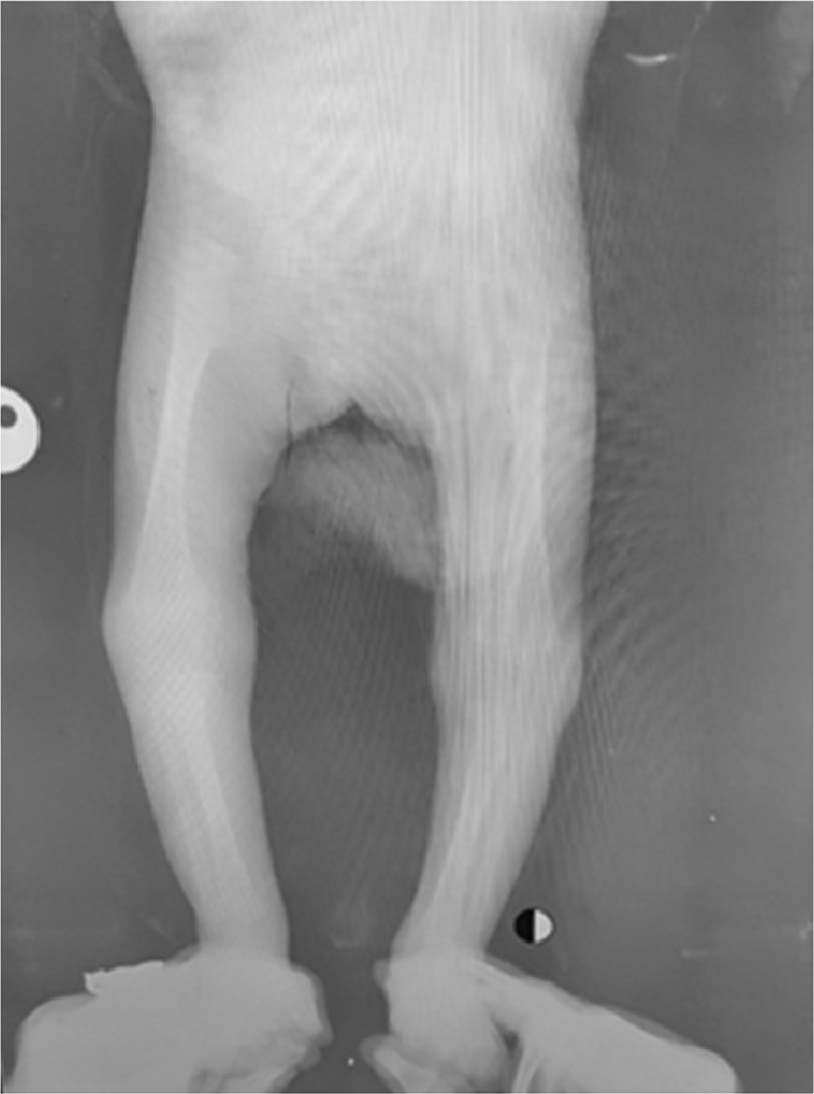
Normal X-rays of the lower limbs.

X-rays of the upper limbs demonstrated a bilateral absence of the radii (Fig. 2), whereas the lower limbs were normal (Fig. 3).

Based on these physical findings, the low platelet count, and radiography results, the diagnosis of TAR syndrome was made.

The diarrhea stopped with lactose-free milk, then milk formula stage 1 feeding.

The managing of this case was not completed after discharging the patient under the responsibility of her parents.

## Discussion

TAR syndrome is a rare congenital defect characterized by hypomegakaryocytic thrombocytopenia and bilateral radii aplasia or hypoplasia in the presence of both thumbs^6^.

The incidence of TAR syndrome in medical literature has been reported to be around 0.42/100 000 live births^7^, it is not related to sex. The sex ratio of diagnosed cases was 1:1^6^. Although other studies reported female’s predilection^8^ ( female patient in our case).

Thrombocytopenia, which may be transient, but is present in 100% of cases diagnosed with TAR syndrome^9^ it may be congenital or may develop within the first few weeks to months of life, although our patient developed it at the age of 6 months. In general, thrombocytopenic episodes decrease with age^10^.

The thrombocytopenia that develops in TAR syndrome is Hypomegakaryocytic Thrombocytopenia platelet hypoproduction caused by insufficient numbers of megakaryocytes, the precursors to platelets, in the bone marrow^11^, as it shown in the bone marrow aspiration that has been performed.

The etiology of the Hypomegakaryocytic thrombocytopenia in these patients is hematopoietic stem cells, which do not appropriately respond to thrombopoietin and impaired maturation of the megakaryocyte progenitor cells in the bone marrow^11^. Several studies have attempted to explain the pathophysiology underlying the impaired megakaryopoiesis seen in TAR syndrome; however, to date this is still unclear^6^.

In some cases, the underlying thrombocytopenia can be occur due to CMPA (cow’s milk protein allergy), as seen in our case. The patient was brought in with chronic diarrhea after cow’s milk introduction, for this reason she presented with a low platelets count of 42 000 plt\ml^11^.

The mechanism of the exacerbation is reported to be direct immunoglobulin E (IgE) immune-mediated or secondary to increased GI bleeding (not shown here) due to loss of coagulation proteins^11^. It should be noted that the patient did not have a thrombocytopenic episode prior to the onset of CMPA. As a result, health care providers should avoid cow’s milk in TAR patients to reduce the severity of gastroenteritis and prevent exacerbations of thrombocytopenia.

Patients with TAR syndrome have absent radii with preserved thumbs and fingers bilaterally. To distinguish TAR syndrome from other conditions associated with phocomelia, such as Holt-Oram syndrome and Fanconi anemia, we detect the presence of both thumbs^6^.

The thumbs of affected individuals are found to be held in flexion against the palm and have a concomitant limited functionality^6^.

These patients tend to have a contracture of the joint, and that may cause a flexion and radial deviation of their hands. So that , hands often being at right angles to the forearms^11^, typically seen in our patient.

The diagnosis of TAR syndrome is established with bilateral absent radii, present thumbs, and thrombocytopenia^10^. The diagnosis of TAR syndrome in our index case was made after noticing the presence of thrombocytopenia in the patient, in addition to radiographic findings.

Our patient had the classic physical features of TAR syndrome. However, the expected decrease in platelet counts to levels below 50 000 plt/ml in the first few weeks of life did not take place in our patient, but she showed up with a platelet count of 42 000 plt/ml after suffering from CMPA for 45 days at the age of 6 months.

Significant laboratory findings in TAR syndrome include a platelet count less than 50 000 plt/ml with normal platelet morphology on peripheral blood film examination. Other associated hematological features are eosinophilia, leukocytosis with a left shift^8^, all these features are seen in our case.

TAR syndrome has been known to be associated with multiple nonskeletal abnormalities include cardiac defects )atrial septal defect and VSD) (15%); our patient’s cardiovascular system does not show any abnormalities, gastroenteritis and cow’s milk intolerance (47%), renal malformations (23%), short stature (95%), and hematologic anomalies^7^. Despite these anomalies, thrombocytopenia remains the main cause of death, particularly during the first two years of life, presenting with intracranial hemorrhages^7^.

## Conclusion

Publication of rare syndromes used to depend on shedding light on tricky cases, or discussing the diagnostic obstacles with those syndromes hided by another disease or a prior diagnostic error.

Thrombocytopenia with absent radii syndrome is a congenital rare case with a clear presentation to be diagnosed, but it is important to note it because the aim of such rare cases is not to discuss what is hidden from the doctor’s mind, as usual, but to take care from a series of its complications that are enable to be improved to reduce consequences by supportive care.

Our aim in publishing the current case report is that clinicians treating patients with TAR syndrome will be aware of the myriad of complications which may occur in the other organ systems so that they can promptly diagnose and care of any associated abnormalities.

## Ethics approval and consent to participate

Not applicable.

## Consent for publication

Written informed consent was obtained from the patient’s parents for publication of this case report and any accompanying images. A copy of the written consent is available for review by the Editor-in-Chief of this journal.

## Sources of funding

Not applicable.

## Authors’ contributions

R.A.: contributed to drafting, reviewing and editing; R.O. is the co- first author with R.A. and N.M.: contributed to drafting, reviewing and editing. N.M. is the co-first author with R.A. and R.O.: contributed to drafting, reviewing, editing and corresponding; H.K.: contributed to reviewing and editing; J.M.: contributed to reviewing, editing, and supervising. All authors read and approved the final manuscript.

## Coflicts of interests disclosure

The authors declare that they have no competing interests.

## Data availability statement

Not applicable.

## Research registration unique identifying number (UIN)

1. Name of the registry: Not applicable.
2. Unique Identifying number or registration ID: Not applicable.
3. Hyperlink to your specific registration (must be publicly accessible and will be checked): Not applicable.

## Guarantor

Dr. Jaber Mahmoud is the guarantor.

## Provenance and peer review

Not commissioned, externally peer reviewed.
